# The effect of combined compression and tactile stimulation on ankle somatosensation in a lunar gravity lower limb load analog

**DOI:** 10.3389/fphys.2025.1537889

**Published:** 2025-02-27

**Authors:** Ashleigh Marchant, Jeremy Witchalls, Sarah B. Wallwork, Nick Ball, Gordon Waddington

**Affiliations:** ^1^ Research Institute for Sport and Exercise, University of Canberra, Canberra, ACT, Australia; ^2^ IIMPACT in Health, University of South Australia, Adelaide, SA, Australia

**Keywords:** somatosensation, hypogravity, microgravity, active movement extent discrimination assessment, compression garment, textured insole, cutaneous feedback, tactile sensation

## Abstract

Ankle somatosensation appears to be negatively affected when in simulated hypogravity (gravity, 1 > g < 0). Developing countermeasures to reduce this negative effect is necessary for sensorimotor control as astronauts prepare to explore the Moon. Head-elevated supine lying has been found to be an effective method in simulating the physiological impact of hypogravity by reducing the weight-bearing capacity through the lower limbs. This study investigated whether wearing a combined compression sock with plantar textured sole (compression-tactile sock) is associated with enhanced somatosensory acuity of the lower limbs in a simulated hypogravity environment. Ankle somatosensory acuity was assessed on 55 healthy participants between the ages of 18 and 65 years (female subjects 28, male subjects 27; mean age 41 years ±14). The active movement extent discrimination assessment (AMEDA) was used to assess somatosensory acuity on participants’ non-dominant foot under four conditions: (1) upright standing in barefoot; (2) upright standing wearing compression-tactile socks; (3) simulated hypogravity (head-elevated supine position) in barefoot; and (4) simulated hypogravity (head-elevated supine position) wearing compression-tactile socks. Analysis was conducted for (i) the whole participant group, (ii) high (above-average) performers, (iii) medium (average) performers, and (iv) low (below average) performers. It was hypothesized that low performers would experience the greatest gains when wearing the sock, compared to those in barefoot. When assessing the group as a whole, AMEDA scores were significantly reduced in the simulated hypogravity (head-elevated supine) conditions when compared to upright standing conditions (p < 0.001; 3% decline when barefoot; 2.9% decline when wearing the socks). Wearing compression-tactile socks had no effect on AMEDA scores when compared to barefoot (p = 0.173). When analyzed by the performance group, somatosensory acuity was enhanced in the compression-tactile sock condition, when compared to barefoot (upright, p = 0.009, 4.7% increase; head-elevated supine, p = 0.022, 3% increase) in the low performers only. In the medium and high-performer groups, there was no difference between the compression-tactile sock conditions and barefoot conditions (p > 0.05 for all). Compression-tactile socks may be associated with enhanced somatosensory acuity in upright standing and simulated hypogravity for individuals with below-average somatosensory acuity. Further research is warranted to assess the effect of compression-tactile socks in an actual hypogravity environment to determine whether the compression-tactile socks can maintain one’s somatosensory acuity.

## 1 Introduction

Exposure to microgravity (µg; characterized by a sense of weightlessness) is known to have a range of unfavorable side effects on human physiology, including reduced bone density, muscle atrophy, decreased cardiovascular fitness, cerebral cortex reorganization, and reduced cognitive function (Carpenter et al., 2010; Clément et al., 2015; Grabherr and Mast, 2010). Astronauts often struggle with standing and mobilizing independently after time spent in microgravity, which produces a risk of failed missions, injury, or death (Carpenter et al., 2010; Grabherr and Mast, 2010). However, the effects on human physiology after exposure to hypogravity are less researched than microgravity (Shelhamer, 2021). Hypogravity can be defined as gravity less than Earth’s gravity (1 g) but more than microgravity (µg) (Lacquaniti et al., 2017). An example of a hypogravity environment is the Moon, also known as lunar gravity, whereby gravity is at 0.16 g. It remains unclear whether lunar gravity (0.16 g) is sufficient to mitigate the negative effects of microgravity (µg) or if physiological systems will continue to deteriorate similarly to that which occurs in microgravity (µg) (Shelhamer, 2021). With plans for astronauts to land and establish their presence on the Moon in the coming years (BBC, 2022; NASA, 2024), it is crucial to have a sound understanding of how exposure to hypogravity impacts human performance to ensure both astronaut safety and mission success.

Limitations arise during microgravity and hypogravity experimentation due to the novelty and physical challenges of the research area, small sample sizes, and high costs (DiFrancesco and Olson, 2015). Many studies, therefore, aim to mimic the effects of reduced gravity while accommodating for the constraints of Earth’s gravity of 1 g. Bed rest is a commonly used technique for both microgravity and, more recently, hypogravity research (Barr et al., 2016). Bed rest involves participants maintaining a flat supine, or −6° head-down tilt, position on the bed for several days or weeks (Pavy-Le Traon et al., 2007). Bed rest techniques can be beneficial in capturing a broad range of participants efficiently at reduced resource cost compared to alternative simulation methods. However, bed rest studies conducted over several days and weeks are time-consuming and demand a high level of participant compliance and adherence (Barr et al., 2016; Pavy-Le Traon et al., 2007). Alternatively, a simple approach to address acute changes in performance is a shift in body orientation from upright to supine position (Marchant et al., 2020; Barr et al., 2016). Head-elevated bed rest, sometimes referred to as head-elevated tilt, is where the patient lies supine with their head elevated and experiences only partial weight bearing longitudinally through the lower limbs to simulate the sensation of hypogravity through the lower limbs and soles of the feet (Barr et al., 2016). Short-term head-elevated tilt experiments are easy to administer and require less participant involvement compared to long-term bed rest studies. Short-term head-elevated tilt protocols can provide insights into how the human body and sensory systems might adjust to acute exposure to hypogravity, thereby providing countermeasures to reduce any unwanted and harmful effects.

The somatosensory system comprises joint position sense (i.e., proprioception) in combination with tactile sensation (i.e., touch). Our bodies continuously receive feedback from the peripheral nervous system (afferent signals) regarding information about the environment. This information is processed by the central nervous system, which interprets it and generates an appropriate motor response (efferent signal) (Riemann and Lephart, 2002a; Riemann and Lephart, 2002b). As an element of the afferent system, the somatosensory system works together with information from the vestibular system (inner ear) and visual system, enabling the brain’s motor centers to maintain an upright posture and stable balance (Park et al., 2023). The somatosensory system consists of three major components: (1) peripheral sensory receptors (e.g., muscle spindles and Golgi tendon organs), (2) ascending pathways, and (3) the somatosensory cortex. Each component plays a vital role in detecting and responding to our surroundings, which, in turn, modulates our movement (Shumway-Cook, 2011). Maintaining optimal somatosensory acuity is, therefore, important for astronauts’ balance and mobility. Any modifications to the nervous system may affect the somatosensory system and influence movement patterns (Barss et al., 2018). If astronauts experience difficulty mobilizing and have poor balance during an emergency evacuation of the spacecraft, this may result in the astronaut being unable to quickly evacuate a capsule to reach safety (Grabherr and Mast, 2010).

Previous research has shown immediate changes to occur to the somatosensory system when exposed to simulated microgravity (µg). These changes included reduced accuracy on ankle somatosensory acuity and altered patterns of upper limb joint position sense, as compared to when upright and full weight bearing (1 g) (Gallagher et al., 2021; Marchant et al., 2020). However, there is limited research focusing on potential changes in the somatosensory system when in hypogravity. Our previous work found that ankle somatosensory acuity was reduced in healthy adults when lying supine head-elevated at 9.6° in a semi weight-bearing position (the lunar wedge bed). The experimental setup mimicked the conditions of 0.16 g (i.e., simulated lunar gravity, 0.16 g) under the feet compared to upright standing in Earth gravity (Marchant et al., 2024a). Other studies on balance and locomotion have shown that humans prefer ‘bouncing’ or ‘skipping’ style gait, compared to conventional walking when in simulated lunar gravity (0.16 g) compared to Earth gravity, which suggests a disturbance to the sensory and motor systems (Pavei and Minetti, 2016). Improving our understanding of the effects of hypogravity environments on lower limb somatosensory changes could help improve the development of interventions that aim to mitigate the negative changes when in hypogravity environments.

A potential intervention to immediately improve lower limb somatosensory acuity may be wearing a textured insole and/or compression garment over the lower leg and foot. It has been suggested that additional tactile stimulation may modulate the somatosensory signal to the central nervous system, thereby providing more feedback and enhancing movement perception (Barss et al., 2018). A textured insole has been shown to provide immediate improvements in lower limb somatosensory acuity, decrease postural sway, and improve postural balance (Kenny et al., 2019; Steinberg et al., 2016a; Steinberg et al., 2016b; Waddington and Adams, 2003). Lower limb compression garments have also been shown to increase one’s ability to detect small changes in the joint movement range of motion (Broatch et al., 2021; Chang et al., 2022). However, more recent data reveal that additional sensory feedback may only be beneficial for individuals with a poor baseline somatosensory ability, with limited benefit to participants with a higher somatosensory ability, and may not necessarily translate to improve postural stability among healthy adults (Broatch et al., 2021; Marchant et al., 2023b; Marchant et al., 2024b). This is potentially important for astronauts as their somatosensory system is likely to be dampened after exposure to hypogravity (Pavei and Minetti, 2016). Furthermore, a systematic review by Woo et al. (2017) suggested that a combination of two or more modalities might be the key to increasing somatosensory stimulation enough to improve overall postural control and balance. A combination of a textured sole and compression of the foot and lower leg may provide a simple approach to improve the proposed lower limb somatosensory acuity reduction when exposed to hypogravity or simulated hypogravity environments.

The primary aim of the current study was to investigate the effects of a compression-tactile sock (combined compression and textured inner sole) on ankle somatosensory acuity in healthy adults under two weight-bearing conditions; (a) Earth gravity, 1 g (upright standing) and (b) a lunar gravity simulation, 0.16 g (head-elevated supine position using a custom-built lunar wedge bed). Previous studies have shown that the effects of compression garments and textured in-soles, compared to barefoot, can be influenced by the participants’ baseline ankle somatosensory acuity. Specifically, individuals with lower somatosensory acuity seem to improve their acuity while wearing these products whereas those with higher somatosensory acuity do not (Marchant et al., 2023b). Prior ankle somatosensory research has achieved this by dividing the group into ‘high’ and ‘low’ somatosensory performer groups (Broatch et al., 2021), or into ‘high,’ ‘medium,’ and ‘low’ tertiles of somatosensory acuity performance (Steinberg et al., 2016b). Therefore, a secondary aim of this study was to further investigate the relationship between compression-tactile socks and somatosensory acuity in two ways; (i) determine whether baseline performance (i.e., barefoot somatosensory acuity while upright standing) is correlated with how much a participant’s ankle somatosensory acuity changes when wearing the compression-tactile socks compared to being barefoot and (ii) whether the effects of the compression-tactile socks gradually decline with greater baseline somatosensory acuity by dividing the group into ‘high,’ ‘medium,’ and ‘low’ somatosensory acuity groups. Our hypothesis was that ankle somatosensory acuity would improve for the whole group while wearing the compression-tactile socks compared to barefoot, with the greatest improvement observed in the lunar wedge bed condition (when ankle somatosensory acuity is reduced) versus upright standing. Additionally, we hypothesized that participants’ baseline performance (barefoot and upright) would correlate with the amount of change in somatosensory acuity from wearing the compression-tactile sock. Specifically, we expected the ‘low’-performer group to show the greatest improvement in somatosensory acuity, while the ‘medium-’ and ‘high’-performer groups would show smaller gains, with the least improvement seen in the ‘high-’performer group.

## 2 Materials and methods

### 2.1 Participants

Fifty-five participants were recruited and completed the study. Demographics of the study population are shown in Table 1. Based on our previous simulated microgravity (µg) study, which showed a medium effect size of 0.69, we used data analytic software G*Power 3.1 (RRID:SCR_013726) to determine that we required at least 54 participants to achieve a statistical power of 0.80 (Marchant et al., 2020). This study was approved by the University of Canberra Human Research Ethics committee (reference number: 202312043). The study protocol was uploaded to the Open Science Framework prior to data collection (Available from osf. io/p8usy) (Marchant et al., 2023a). Inclusion criteria included adults between 18 and 65 years who considered themselves healthy and unrestricted. This was to ensure they could complete the somatosensory acuity task and move between positions without difficulty. Exclusion criteria included any medical conditions which might affect balance (such as diminished tactile sensation or inner ear function) or any ankle injury within the previous 3 months.

**TABLE 1:** CAI, chronic ankle instability; Hx, history; M, mean; SD, standard deviation; Sex: F, female; M, male.

Characteristic	
Sex	F 28 M 27
Age (M ± SD)	41 years ±14
Height (M ± SD)	173 cm ± 9
Body mass (M ± SD)	76 kg ± 9
Foot dominance	R 49 L 6
Hx of ankle injury in testing foot	14 (+11 uncertain)
CAIT questionnaire (M ± SD)	26 ± 5
Likely to have CAI	14

### 2.2 Ankle somatosensory acuity

**FIGURE 1:**
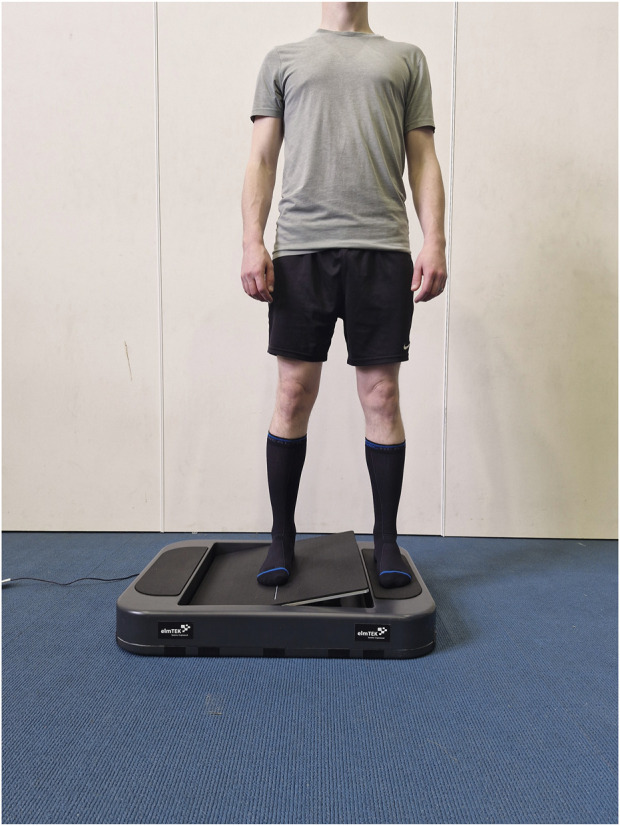
Participant wearing the combined compression-tactile socks and completing the active movement extent discrimination assessment (AMEDA) with their right foot on the testing platform. During the task, participants were asked to move the platform (testing foot) by inverting their right ankle as far as the platform would allow (rigid endpoint) to one of five pre-defined depths.

Ankle somatosensory acuity was assessed using an active movement extent discrimination assessment (AMEDA) protocol (Figure 1). The AMEDA requires participants to make an absolute judgment about the joint position in an active manner using proprioceptive feedback (Han et al., 2016). The ankle AMEDA used in this study required participants to stand on a platform that had a stationary plate (for their non-testing foot) and a moveable plate (for their testing foot). The moveable plate tilted in the frontal plane into five possible predetermined depths of ankle inversion. Participants were required to make an absolute judgment about which position (depth of inversion) they had experienced, thereby assessing their ability to discriminate between small changes in the ankle range of movement. Position 1 was 10.5° of ankle inversion (i.e., relative to the horizontal). Each position then increased by 1° further into ankle inversion, with position 5 being 14.5° of ankle inversion. To familiarize participants with the five positions, participants were exposed to the five depths of ankle inversion in a sequential order, three times prior to the commencement of the formal assessment. Participants were asked to move the plate into ankle inversion until they reached the end point (i.e., the platform stopped) and then return to the horizontal neutral position. After familiarization, formal testing commenced. Participants were presented with 50 ankle inversion depths in a pseudorandom order and were required to respond verbally with the position (1–5) they thought they had experienced after each movement. The order was pseudorandomized, and each position was presented 10 times. Participants were always in control of the movements and were asked to maintain their gaze straight ahead (or perpendicular to their body for the supine condition) to reduce feedback from visual cues. The assessment was completed on the participants’ non-dominant (non-kicking) foot only. When assessing ankle somatosensory acuity on AMEDA, accuracy on the non-dominant foot has been shown to be higher than on the dominant foot (Han et al., 2013).

Participant inversion depth responses were recorded manually by the principal investigator via an Android tablet and uploaded to a Microsoft Excel spreadsheet (Microsoft® Corporation. 2023. Version 2301. Retrieved from https://office.microsoft.com/excel). A matrix of responses was generated to form an area under the curve (AUC) score of a receiver operating characteristic (ROC) curve with scores between 0.5 and 1.0. A score closer to 0.5 indicated chance, and a score of 1.0 indicated perfect accuracy. The ankle AMEDA has shown to have good test–retest and intra-rater reliability in healthy adults (ICC: 0.80), although reliability can be reduced in individuals with ankle dysfunction, for example, chronic ankle instability reduces the reliability to moderate reliability (ICC: 0.60) (Shi et al., 2023; Witchalls et al., 2014).

### 2.3 Lunar wedge bed

**FIGURE 2:**
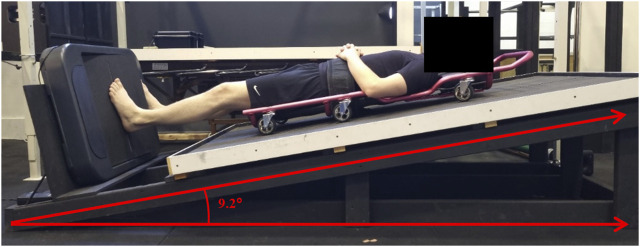
Participant lying supine on the lunar wedge bed with their feet upon the ankle AMEDA and their head elevated at 9.2° from the horizontal position. In this orientation, participants had approximately 16% of their body weight loaded through their plantar feet. Image reprinted with permission from Marchant et al. (2024a).

A lunar wedge bed was constructed for the purpose of this research (Figure 2). The lunar wedge bed was a timber truss-style system with a timber board positioned 9.2° above the horizontal position. Participants were required to lie (supine) upon a traditional mechanics’ creeper, which had six wheels, on top of the timber board. The ankle AMEDA was positioned at the base of the wedge bed, perpendicular to the timber board, so that when participants were supine, they could position their feet onto the ankle AMEDA, and the traditional mechanics’ creeper reduced friction under their torso. In this position with their head elevated, participants had approximately 16% of their body weight loaded through the plantar aspect of their feet. This technique was used to mimic the pressure of lunar gravity under the feet, equivalent to 0.16 g. The effects of the lunar wedge bed on ankle somatosensory acuity, muscle activity, and muscle characteristics when barefoot have been described previously (Marchant et al., 2024a).

### 2.4 Tactile conditions: barefoot and compression-tactile sock

**FIGURE 3:**
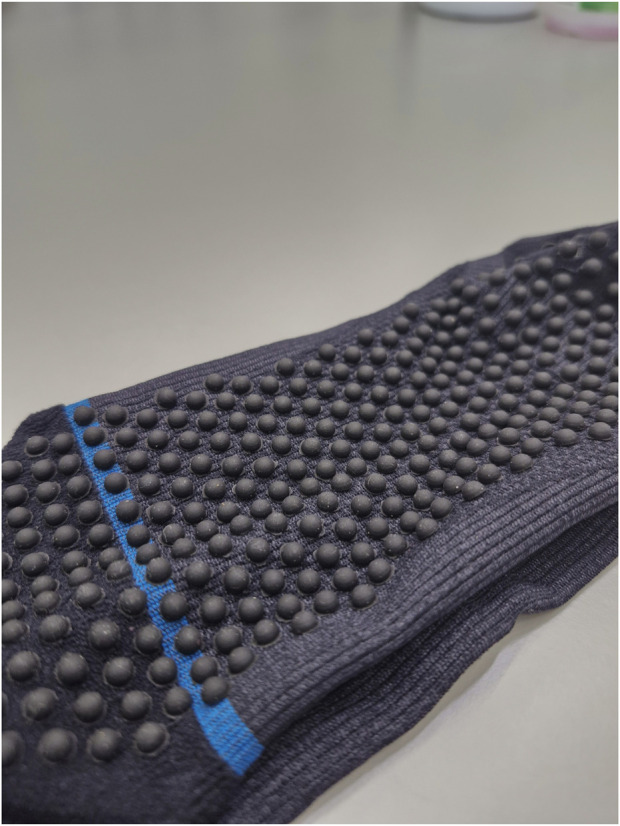
Compression-tactile socks had small nodules attached to the inside of the sole of the foot. The sock is turned inside-out for the photo.

Ankle somatosensory acuity was assessed in upright standing (Earth gravity, 1 g) and head-elevated supine lying (simulated lunar gravity, 0.16 g) under two tactile conditions (1) barefoot and (2) wearing mixed compression-tactile socks (Figure 3). The socks were provided by SRC Health Pty Ltd. for the purpose of this research. When worn, the socks reached approximately 50 mm below the head of the fibula and provided a compression rate of 20–30 mmHg. In addition to the compression, small rubber nodules were attached to the inside of plantar aspect of the sock, with the rubber nodules being in direct contact with the plantar sole of the foot.

### 2.5 Procedure

Participants attended the laboratory for a single 60-minute session. Written informed consent was obtained prior to participation. Demographic data were collected on participants’ age, sex (as assigned at birth), height, weight, previous ankle injury, and preferred kicking foot. Participants completed a Cumberland Ankle Instability Tool (CAIT) questionnaire to assess their concurrent level of ankle instability (Hiller et al., 2006). Participants then completed the familiarization process on the AMEDA in upright standing, followed by four sets of ankle AMEDAs under the four conditions in a randomized block design. The four conditions are as follows:

(i) Upright standing (Earth gravity, 1 g) barefoot.
(ii) Upright standing (Earth gravity, 1 g) wearing the compression-tactile sock.
(iii) Supine lying on the lunar wedge bed (simulated lunar gravity, 0.16 g) in barefoot.
(iv) Supine lying on the lunar wedge bed (simulated lunar gravity, 0.16 g) wearing the compression-tactile sock.

After each condition, participants were instructed to remove their sock (if they were wearing one) and walk across the room (approximately 20 m) to ‘reset’ between tests.

### 2.6 Statistical analysis

Demographic data, CAIT questionnaire results, and four ankle AMEDA AUC scores representing the four conditions from each participant were calculated for analysis. SPSS statistics (IBM Corp. Released 2023. IBM SPSS Statistics for Windows, Version 29.0. Armonk, NY: IBM Corp) was used to analyze all results, with an alpha value of 0.05 to represent statistically significant results. Data were tested for normality via a Shapiro–Wilk test. Visual inspection of a boxplot for values greater than 1.5 box-lengths from the edge of the box was used to identify outliers. Due to the large age range, a one-way analysis of variance (ANOVA) was conducted to assess whether there was any change in AMEDA AUC barefoot upright scores among age groups (i.e., group ‘a’: 18–29 years; group ‘b’: 30–39 years; group ‘c’: 40–49 years; group ‘d’: 50–59 years; and group ‘e’: 60–65 years). A one-way repeated-measures ANOVA was conducted to assess whether there was any change in AMEDA AUC scores among the sequence of ankle AMEDA tests that might signify a learning effect (i.e., if somatosensory acuity improved after each assessment, regardless of condition). An independent t-test with Bonferroni adjustment was conducted to assess whether there was a difference in ankle AMEDA AUC scores between those with and without chronic ankle instability (CAI), as determined by the CAIT results.

#### 2.6.1 AMEDA analysis: whole group

To address our primary aim of assessing the effects of a compression-tactile sock on ankle somatosensory acuity in healthy adults under two weight-bearing conditions of (a) Earth gravity, 1 g (upright standing), and (b) a lunar gravity simulation, 0.16 g (head-elevated supine on the lunar wedge-bed), a 2 × 2 repeated-measures ANOVA was conducted (two levels of position: upright standing and lunar wedge bed; two levels of tactile condition: barefoot and compression-tactile socks). *Post hoc* paired t-tests were conducted to further explore any significant effects.

#### 2.6.2 AMEDA analysis: high, medium, and low performers

To address our secondary aim of (i) determining whether baseline performance (i.e., barefoot somatosensory acuity whilst upright standing) is correlated with how much a participant’s ankle somatosensory acuity changes when wearing the compression-tactile socks compared to being barefoot and (ii) whether the effects of the compression-tactile socks gradually decline with greater baseline somatosensory acuity by dividing the groups into ‘high,’ ‘medium,’ and ‘low’ somatosensory acuity groups. For further investigating the relationship between compression-tactile socks and somatosensory acuity by dividing participants into ‘low’, ‘medium’, and ‘high’ performance groups, as based on their baseline somatosensory performance, two analyses were conducted.

To address item (i), we conducted a Pearson’s correlation for two comparisons:

(1) the correlation between baseline (1 g) barefoot AMEDA AUC scores and the amount of change in *barefoot* AMEDA AUC scores from standing (Earth gravity, 1 g) to laying supine on the lunar wedge bed (lunar gravity, 0.16 g);
(2) the correlation between baseline (1 g) barefoot AMEDA AUC scores and the amount of change in *compression-tactile socks* AMEDA AUC scores from standing (Earth gravity, 1 g) to laying supine on the lunar wedge bed (lunar gravity, 0.16 g).

The strength of the relationship was determined as described by Cohen (1992), where correlation coefficients of 0.10, 0.30, and 0.50 were considered to be small, medium, and large in magnitude, respectively.

To address item (ii), a 3 × 2 × 2 repeated-measures ANOVA was conducted (three performance levels: ‘high,’ ‘medium,’ and ‘low’; two levels of body orientation: upright standing and lying on a lunar wedge bed; two levels of tactile conditions: barefoot and wearing compression-tactile socks). Participants were first grouped into tertiles based on the score they obtained during the upright standing (1 g) condition on barefoot. This was calculated so that we had approximately equal sample sizes for each performance group. Previous ankle AMEDA studies have also employed this technique, aiming to reduce the effects of skewed data and unequal group sample sizes (Broatch et al., 2021; Steinberg et al., 2016a). Participants were therefore grouped as ‘high,’ ‘medium,’ and ‘low’ performers. *Post hoc* paired t-tests were conducted to further explore any significant effects.

## 3 Results

### 3.1 Participant characteristics

Data were normally distributed for all conditions (p > 0.05) except for the upright (Earth gravity, 1 g) compression-tactile sock condition (p = 0.003). This was due to one outlier who scored poorly in the upright compression-tactile sock condition. However, this participant was retained in the analysis as it was considered a genuine but unusual data point and was consistent with this participants’ other scores. There were no statistically significant differences in AMEDA AUC scores between the different age groups (F (4, 50) = 0.703, p = 0.593).

Participants’ mean AMEDA AUC score of their first test (regardless of condition) was 0.679, followed by 0.676 for their second, 0.678 for their third, and 0.696 for their fourth. The one-way repeated-measures ANOVA revealed a significant change among this sequence of ankle AMEDA tests (F (3, 162) = 3.73, p = 0.013). *Post hoc* analysis with a Bonferroni adjustment revealed that there was a significant increase in AMEDA AUC scores of 0.02 between the second and fourth AMEDA (p = 0.014), regardless of the condition.

There was no significant difference between AMEDA AUC scores between those with and without CAI for the upright standing (Earth gravity, 1 g) barefoot condition (p = 0.178), upright standing (Earth gravity, 1 g) compression-tactile sock condition (p = 0.019), lunar wedge bed (simulated lunar gravity, 0.16 g) barefoot condition (p = 0.175), or lunar wedge bed (simulated lunar gravity, 0.16 g) compression-tactile sock condition (p = 0.078).

### 3.2 AMEDA results: whole group analysis

**FIGURE 4:**
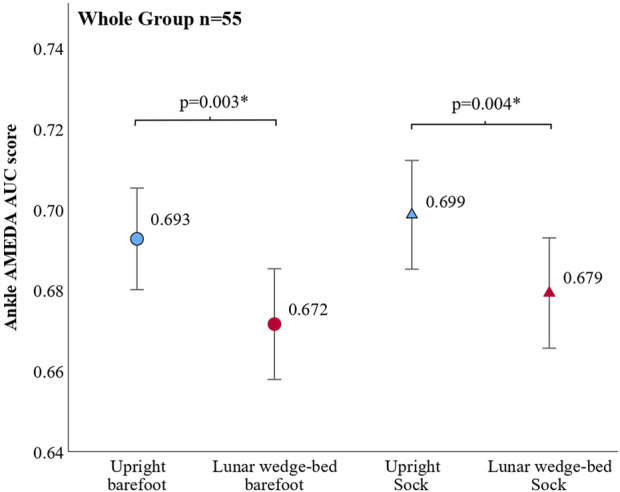
Results of ankle AMEDA AUC scores for the whole group. Responses are represented as an AUC score between 0.5 (chance) and 1.0 (perfect score). Somatosensory acuity decreased when on the lunar wedge-bed (simulated lunar gravity, 0.16 g) compared to upright standing (Earth gravity, 1 g) for both barefoot and when wearing the compression-tactile socks. There was no significant difference between barefoot and compression-tactile socks for both body orientations. AMEDA AUC score means are shown with pairwise comparison, where * denotes a significant result. Error bars represent 95% confidence intervals. Results suggest that although somatosensory acuity was reduced on the lunar wedge bed compared to the upright position, the socks were ineffective in improving acuity, thus rejecting our primary hypothesis.

When the whole group was analyzed, there was no main effect of tactile condition (compression-tactile sock versus barefoot) (F (1,54) = 1.91, p = 0.173). There was a main effect of body orientation (upright standing versus lunar wedge bed) (F (1,54) = 15.68, p < 0.001) such that AMEDA AUC scores were lower when on the lunar wedge bed than when upright standing. There was no significant interaction between body orientation and the tactile condition (F (1,54) = 0.04, p = 0.839). *Post hoc* analysis with a Bonferroni adjustment revealed that there was a significant decrease in AMEDA AUC scores when on the lunar wedge bed compared to the upright standing in barefoot condition (p = 0.004, 3% decline) and compression-tactile sock condition (p = 0.03, 2.9% decline). AMEDA AUC score means, confidence intervals, and pairwise comparisons are presented in Figure 4.

### 3.3 AMEDA results: high, medium, and low performer group analysis

To conduct Pearson’s correlation for item (i) and whether AMEDA baseline performance (i.e., barefoot somatosensory acuity when upright standing) is correlated with how much a participant’s ankle somatosensory acuity changes when wearing the compression-tactile socks compared to being barefoot, the difference in AMEDA AUC scores was first calculated for the following:

(1) the amount of change in barefoot AMEDA AUC scores from standing (Earth gravity, 1 g) to laying supine on the lunar wedge bed (simulated lunar gravity, 0.16 g) and
(2) the amount of change in compression-tactile sock AMEDA AUC score from standing (Earth gravity, 1 g) to laying supine on the lunar wedge bed (simulated lunar gravity, 0.16 g).

The amount of change between postures when barefoot was 0.021, while the amount of change between postures when wearing socks was 0.013. The correlation analysis exposed a significant, medium Pearson’s correlation between the baseline (barefoot) amount of change in AMEDA AUC score from standing to laying supine on the lunar wedge bed for the barefoot condition, and a significant, large Pearson’s (Cohen, 1992) correlation for the compression-tactile condition (r (53) = 0.471, p < 0.001, and r (53) = 0.523, p < 0.001, respectively).

To conduct the 3 × 2 × 2 repeated-measures ANOVA for item (ii) and to assess whether the effects of the compression-tactile socks gradually decline with greater baseline somatosensory acuity, participants were categorized into three groups based on their baseline barefoot AMEDA performance. Participants were categorized with approximately equal sample sizes across the three groups, rather than grouping based on absolute AMEDA AUC values. Group 1 included participants who had an upright standing (Earth gravity, 1 g) barefoot AMEDA AUC score under 0.68 (n = 19) and were considered ‘low’ performers. Group 2 included participants who had an upright standing (Earth gravity, 1 g) barefoot AMEDA AUC score between 0.68 and 0.709 (n = 19) and were considered ‘medium’ performers. Group 3 included participants who had an upright standing (Earth gravity, 1 g) barefoot AMEDA AUC score of 0.71 and above (n = 17) and were considered ‘high’ performers. There was a significant main effect on performance groups (low versus medium versus high) (F (2,52) = 20.22, p < 0.001, ε = 0.437) such that those in the low performing group had the lowest AMEDA AUC scores, while those in the high performing group had the highest AMEDA AUC scores. There was a significant main effect on body orientation (upright standing versus lunar wedge bed) (F (2, 52) = 0.747, p < 0.001) such that AMEDA AUC scores were lower when on the lunar wedge bed (simulated lunar gravity, 0.16 g) than when upright standing (Earth gravity, 1 g). There was no main effect of tactile condition (compression-tactile sock versus barefoot) (F (2, 52) = 0.967, p = 0.186). There was no significant interaction between performance group, tactile condition, and body orientation (F (2,52) = 0.972, p = 0.479).

**FIGURE 5:**
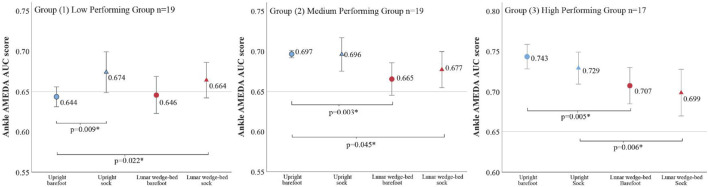
Pairwise comparison results (indicated by p-values shown) of ankle AMEDA AUC scores when analyzed by the performance group. In the low-performer group (left panel), somatosensory acuity was unaffected by the change in body orientation from upright (Earth gravity, 1 g) to lying on the lunar wedge bed (simulated lunar gravity, 0.16 g). Wearing compression-tactile socks improved somatosensory acuity in this group. The medium- and high-performer groups (middle and right panel) did not gain by wearing the compression-tactile socks; however, they were negatively impacted by the change in body orientation. AMEDA AUC score means are shown with pairwise comparison, where * denotes the significant result. Error bars represent 95% confidence intervals. The horizontal reference line at AUC score 0.65 represents a proposed “cut-off,” of where AMEDA scoring under this line is associated with injury (discussed below). Note: a change in scale within the high-performing group (right panel).

In the low performing group, there was a significant main effect on tactile condition (F (1,18) = 7.55, p = 0.013) but not body orientation F (1,18) = 0.29, p = 0.596. There was no significant interaction between body orientation and the tactile condition for AMEDA AUC scores (F (1,18) = 0.56, p = 0.463). *Post hoc* t tests revealed that there was a significant increase in AMEDA AUC scores when wearing the compression-tactile sock compared to barefoot (in an upright position t (18) = 2.62, p = 0.009, 4.7% increase) but no significant difference in AMEDA AUC scores when wearing the compression-tactile socks compared to barefoot when on the lunar wedge bed (t (18) = 1.50, p = 0.076). Participants’ highest AMEDA AUC score in this performance group was when standing upright and wearing the compression-tactile sock (AUC: 0.67). However, their next best score was when on the lunar wedge bed and wearing the compression-tactile sock (AUC: 0.66), and this score was significantly higher than the AMEDA AUC score in the upright barefoot (AUC: 0.64) condition (t (18) = 2.17, p = 0.022, 3% increase), suggesting that the socks increased somatosensory acuity, while body orientation had no influence in people with low baseline somatosensory performance. AMEDA AUC score means, confidence intervals, and pairwise comparisons are presented in Figure 5.

In the medium performing group, there was a significant main effect on body orientation (F (1,18) = 7.20, p = 0.015), but there was no main effect on tactile condition (F (1,18) = 0.64, p = 0.435). There was no significant interaction between body orientation and tactile condition for AMEDA AUC scores (F (1.18) = 0.85, p = 0.368). *Post hoc* t-tests revealed that when barefoot, there was a significant decrease in AMEDA AUC scores when on the lunar wedge bed compared to when upright (t (18) = 3.17, p = 0.003, 4.6% decline). However, when wearing the compression-tactile socks, there was no significant difference between the lunar wedge bed and upright AMEDA AUC scores (t (18) = 1.46, p = 0.080). There was a significant decrease in AMEDA AUC scores when on the lunar wedge bed and wearing the compression-tactile socks, compared to when upright and barefoot (t (18) = 1.79, p = 0.045, 2.9% decline), suggesting that somatosensory acuity was poorer when on the lunar wedge-bed even with the additional stimulation from the socks. Participants in this group had mean AMEDA AUC upright scores almost identical for both tactile conditions (AUC: 0.697 and 0.696 for barefoot and sock, respectively). AMEDA AUC score means, confidence intervals, and pairwise comparisons are presented in Figure 5.

In the high performing group, there was a significant main effect on body orientation (F (1,16) = 14.00, p = 0.002), but there was no main effect on the tactile condition (F (1,16) = 2.11, p = 0.166). There was no significant interaction between the body orientation and tactile condition for AMEDA AUC scores (F (1,16) = 0.13, p = 0.722). *Post hoc* t-tests revealed that there was a significant decrease in AMEDA AUC scores when on the lunar wedge bed compared to upright standing on barefoot (t (16) = 2.93, p = 0.005, 4.8% decline) and when wearing the compression-tactile socks (t (16) = 2.86, p = 0.006, 4.1% decline). Participants’ best score in this group was when they were upright and barefoot (AUC: 0.74), while their lowest score was when on the lunar wedge bed and wearing the compression-tactile socks (AUC: 0.70). AMEDA AUC score means, confidence intervals, and pairwise comparisons are presented in Figure 5.

## 4 Discussion

The aim of this study was to investigate the effects of compression plus tactile stimulus on ankle somatosensory acuity in healthy adults under two weight-bearing conditions; (a) Earth gravity, 1 g (upright standing) and (b) a lunar gravity simulation, 0.16 g (head-elevated supine position using a custom-built lunar wedge bed). Results showed that when participants were analyzed as a whole group, ankle somatosensory acuity was reduced when on the lunar wedge bed (simulated lunar gravity, 0.16 g) compared to when upright (Earth gravity, 1 g). However, there was no increase in performance when participants wore the compression-tactile socks than when compared to barefoot. When participants were grouped according to their baseline AMEDA AUC scores, those classified as ‘low’ performers displayed enhanced somatosensory acuity when wearing the compression-tactile socks. In contrast, both ‘medium-’ and ‘high’-performer groups demonstrated reduced performance in the lunar wedge bed condition when compared to upright standing but had no change in performance when wearing the compression-tactile sock. Overall, this suggests that the compression-tactile sock might have some benefits for individuals with low baseline somatosensory acuity.

Assessed as the whole group, somatosensory acuity was reduced on the lunar wedge bed (simulated lunar gravity, 0.16 g) condition compared to upright standing, which aligns with previous microgravity (µg) research. Astronauts exposed to microgravity (µg) often experience disruptions in all their sensory systems. As a result, astronauts appear to rely more on somatosensory information for postural balance and movement control compared to individuals who have not been exposed to microgravity (µg), particularly while their vestibular system adjusts (Ozdemir et al., 2018). This suggests that the integration of sensory afferent signals may vary within the central nervous system when body weight is modified, whether it be in µg conditions, or under simulated 0.16 g, as observed in the current study. Macaulay et al. (2021) suggested targeting interventions at the somatosensory system may be crucial for improving balance after microgravity (µg) exposure. However, when assessed as the whole group, the current study reveals that the compression-tactile socks, which target the somatosensory system, were ineffective at improving somatosensory acuity in both upright (Earth gravity, 1 g) and lunar wedge bed (simulated lunar gravity, 0.16 g) conditions. Furthermore, our previous work has shown that the compression-tactile socks were not beneficial at improving postural stability compared to being barefoot under Earth (1 g) conditions (Marchant et al., 2024b). This suggests that among healthy adults, there is variability in how effective the socks can be on somatosensory acuity, and any improvements do not necessarily translate over to postural stability. Alternatively, it indicates that the central processing of sensory input is also variable depending on the task or body weight status. Further research is warranted to explore interventions aimed at stimulating the somatosensory system, with a focus on the underlying neurophysiological mechanisms, and the effectiveness of the compression-tactile socks under true hypogravity conditions.

With further analysis, the compression-tactile socks were shown to be beneficial for improving ankle somatosensory acuity in individuals with low somatosensory acuity, relative to this participant sample but with no notable effect for those in the medium- and high-performance groups. Although the average somatosensory scores are not an indication of what might be considered average scoring for the broader population, these findings might have implications for those with medical conditions, musculoskeletal injury, or astronauts, whose somatosensory functioning is likely to be poor (Haslam et al., 2023; Macaulay et al., 2021; Witchalls et al., 2014). Previous research indicates that ankle injury and ligament instability are associated with low ankle AMEDA AUC scores and that a score of 0.65 and below can reflect an athlete’s readiness to return to sport after ankle injury (Steinberg et al., 2019; Witchalls et al., 2013; Stokes et al., 2020). In our study, we recruited individuals who considered themselves healthy and unrestricted, and so our categorization on the performance level was relative to within the cohort, rather than using an absolute AUC value or cut-off score based on a pathological condition. We did, however, include individuals with possible CAI. Based on CAIT results, there was no significant difference in AMEDA AUC scores between those with and without CAI. Future research should focus on evaluating the impact of compression-tactile socks on ankle somatosensory acuity in other populations and not just among healthy adults, as ours has.

Low ankle somatosensory acuity is associated with poor postural stability among healthy adults (Marchant et al., 2024c) and increased fall risk among older adults (Antcliff et al., 2023). Although the current study did not focus on these risk factors, it is evident that poor somatosensory acuity is undesirable. In the current study, low-performing participants improved their AMEDA AUC with compression-tactile socks in both the upright (Earth gravity, 1 g) and lunar wedge bed (simulated lunar gravity, 0.16 g) conditions. Notably, low-performing group participants’ lunar wedge bed compression-tactile sock scores were significantly higher than their barefoot upright AMEDA AUC score, indicating the socks enhanced somatosensory acuity despite a loss of sensation from the reduced weight bearing when on the lunar wedge bed. Interestingly, the barefoot upright AMEDA AUC score is below the 0.65 AUC score previously associated with ankle injury and ligament instability (Steinberg et al., 2019; Stokes et al., 2020; Witchalls et al., 2013), and wearing the compression-tactile sock improved the score, raising it above the 0.65 threshold. For astronauts exposed to long term hypogravity or microgravity, where their sensory systems are compromised, textured socks could be a practical solution to immediately improve somatosensation and perhaps assist in overall mobility.

In contrast to the low-performer group, participants in the medium- and high-performer groups did not benefit from wearing the compression-tactile socks but were affected by changes in orientation or weight-bearing. A lack of improvement to lower limb somatosensory acuity with a garment among high performers has been observed in other ankle AMEDA studies (Broatch et al., 2021; Marchant et al., 2023b). This suggests that some individuals may already receive sufficient sensory input from their somatosensory system, leading to a ceiling effect, where additional external feedback from the garment is not utilized effectively. This could also explain why these participants were impacted by the shift from upright (Earth gravity, 1 g) to the lunar wedge bed (simulated lunar gravity, 0.16 g) condition. In a full weight-bearing position, sensory feedback is robust, and all postural muscles are activated. However, muscle activity is diminished when in a semi-weight bearing position, thereby having a significant impact on the available sensory feedback and proprioception that the participant receives (Marchant et al., 2024a). We hypothesized that the lunar wedge bed condition would impair somatosensory performance enough to allow the external feedback from the compression-tactile socks to compensate; however, results did not support this. It appears that the change in posture and the associated reduced weight bearing affect high performers’ ability to integrate somatosensory feedback from muscle inputs and proprioception; however, no additional benefit is achieved from the tactile aspect of their somatosensation, resulting in the compression-tactile socks being ineffective. However, it remains uncertain if high-performing individuals were exposed to a reduced weight-bearing environment for an extended period, such as mobilizing on the Moon in 0.16 g, or whether this would lead to further degradation of somatosensory acuity. If this did occur, it could result in a potential shift of reliance from internal to external sensory feedback, making the socks more advantageous. Future research could investigate the impact of prolonged bed rest on somatosensory acuity to explore this possibility.

When the whole group was analyzed, the beneficial effect of wearing the compression-tactile sock in the low-performing group appeared to be diluted by the lack of response from the medium- and high-performer groups, and only the change in posture appeared to have a significant effect. The significant Pearson’s correlations, however, revealed a linear relationship in the changes (for both barefoot and wearing the compression-tactile socks), indicating a meaningful relationship between barefoot somatosensation in standing and the amount of change that was occurring between the two postures. This highlights the value of segmenting the sample and examining individual responses to the different conditions. Although both the medium- and high-performer groups did not benefit from wearing the compression-tactile socks and instead showed decreased AMEDA AUC scores in the lunar wedge bed condition compared to upright standing, the changes in the medium-performer group were more subtle than those in the high-performer group. In the medium-performer group, AMEDA AUC scores did not significantly differ between the upright (Earth gravity, 1 g) and lunar wedge bed (simulated lunar gravity, 0.16 g) conditions while wearing the compression-tactile sock, indicating that somatosensory acuity was maintained. Examining the medium-performer group AMEDA AUC scores alongside the correlation analysis confirms a linear relationship existing across the population’s AMEDA AUC scores, suggesting a gradual shift in reliance between sock and weight-bearing status. Additionally, the low-performer group exhibited the smallest change in AMEDA AUC scores from upright to the lunar wedge bed. However, the significant, large effect size of the correlation revealed a stronger relationship between baseline scores and changes in AMEDA AUC scores with compression-tactile socks, compared to changes observed when in barefoot. These results indicate that performance on the AMEDA strongly related to the degree of a participant’s responses to changes in weight-bearing or interventions, such as when wearing compression-tactile socks. Therefore, individualized prescription of interventions is crucial for researchers and therapists when assessing the suitability of such interventions, whether it be for astronauts preparing for lunar exploration or for rehabilitation on Earth.

### 4.1 Limitations

This study has several limitations. First, the lunar wedge bed designed for this research presents a simple, yet effective approach to deload the lower limbs, simulating the effects of 0.16 g loading. Although this method provides valuable insights into what might occur to the somatosensory system under such conditions, it is crucial to emphasize this research is a simulation only. The inherent limitation of the design is we cannot eliminate the 1-g vertical vector loading and results should be interpreted within this context.

Second, the improvement in AMEDA AUC scores by the fourth assessment suggests a learning effect to have occurred in that somatosensory acuity was improved through repeated exposure. Although this finding is consistent with previous research (Smyth et al., 2021; Witchalls et al., 2014), we were unsure whether changing the orientation (upright and supine on the lunar wedge bed) would dampen the learning effect. Although participants were pseudorandomized via the different conditions as a whole group, this randomization did not extend to the categorization of performance groups based on the method used. Future research should account for this learning effect as participants may improve their performance on AMEDA testing with short periods between tests, potentially improving their scores even with different interventions (such as socks and orientation).

Third, the age range and exclusion criteria for this study were broad. Although previous research indicates that somatosensory function can decline with age, the AMEDA is mostly influenced by individuals who are very young (i.e., under 10 years) and those aged 75 and above (Yang, et al., 2019), both of which were excluded from this study. Additionally, while participants were deemed healthy and unrestricted in movement, factors such as body mass index, physical activity levels, and medication intake were not controlled for. This did allow for a broad sample of participants to increase generalisability of the findings, but these factors should be considered when interpreting the results.

Finally, when astronauts mobilize in environments such as on the surface of the Moon, they will be wearing a spacesuit, which significantly differs from the current study focused on comparing compression-tactile socks to barefoot conditions. The altered locomotion patterns experienced by astronauts during the Apollo missions may be partly due to the heavy spacesuits that limited knee and hip mobility (Newman et al., 2007; Pavei and Minetti, 2016; Thuro and Stirling, 2021). It is unclear whether the compression-tactile socks would result in different effects had they been compared to a space boot instead of barefoot. Furthermore, the results are limited by the stationary nature of the AMEDA. Although the assessment involves active, participant-driven movement, it is unclear how this might translate to mobility and locomotion. Integrating a better understanding of somatosensory performance in these spacesuits, particularly under real-life mission scenarios, would improve relevance for astronauts preparing for the Moon missions.

## 5 Conclusion

Wearing a combined compression and tactile stimulating sock was associated with better ankle somatosensory acuity compared to barefoot, among healthy adults with low somatosensory acuity, both in an upright, full weight-bearing position, and in a supine lying, reduced weight-bearing position (lunar gravity analog). Compression-tactile socks might improve ankle somatosensory acuity in hypogravity conditions for individuals with poor baseline acuity, although further work is warranted to review the underlying neurophysiological mechanisms contributing to the observed variability, and to assess the effects within a true hypogravity environment. Further research is also needed to explore alternative methods to improve somatosensory acuity for individuals with average or above-average baseline acuity after hypogravity or simulated hypogravity exposure, particularly in sustained mission contexts and to assess whether the compression-tactile socks can maintain one’s somatosensory acuity in low gravity conditions.

## Data Availability

The raw data supporting the conclusions of this article will be made available by the authors, without undue reservation.
